# The Use of the Cryopreserved Aortic Homograft for Aortic Valve Replacement: Is It Still an Option?

**DOI:** 10.3390/jcdd10060248

**Published:** 2023-06-08

**Authors:** Francesco Nappi, Antonio Nenna, Cristiano Spadaccio, Sanjeet Singh Avtaar Singh, Almothana Almazil, Christophe Acar

**Affiliations:** 1 Department of Cardiac Surgery, Centre Cardiologique du Nord, 93200 Saint Denis, France; 2 Department of Cardiovascular Surgery, Università Campus Bio-Medico di Roma, Via Alvaro del Portillo, 00128 Roma, Italy; a.nenna@policlinicocampus.it; 3 Cardiothoracic Surgery, Lancashire Cardiac Center, Blackpool Victoria Hospital, Blackpool FY3 8NP, UK; 4 Department of Cardiothoracic Surgery, Royal Infirmary of Edinburgh, Edinburgh EH16 4SA, UK; sanjeetsinghtoor@gmail.com; 5 Department of Cardiothoracic Surgery, Hôpital Pitié-Salpêtrière, Boulevard de Hôpital 47-83, 75013 Paris, France

**Keywords:** aortic homograft, aortic valve disease, aortic valve replacement, pregnancy, endocarditis

## Abstract

The indications for cryopreserved allografts in aortic valve replacement are still debatable. We aim to identify factors influencing early and long-term durability of the aortic homograft and to define subgroups of patients with an improved long-term quality of life, survival, and freedom from structural valve degeneration (SVD). We evaluated our series of 210 patients who underwent allograft implantation with a retrospective cohort study design over a period of 20 years. Endpoints were overall mortality, cardiac mortality related to SVD, the incidence of SVD, reoperation, and a composite endpoint comprising major adverse cardiac and cerebrovascular events (MACCEs), which includes cardiac death both related and not related to SVD, subsequent aortic valve surgery, new or recurrent infection of implanted allograft, recurrent aortic regurgitation, rehospitalization for heart failure, an increase in New York Heart Association (NYHA) class of ≥1, or cerebrovascular events. The primary indication for surgery was endocarditis (48%), which was also a predisposing factor for increased cardiac mortality. Overall mortality was 32.4% with a 27% incidence of SVD and mortality associated with SVD of 13.8%. Reoperation occurred in 33.8% and MACCEs in 54.8%. Long-term NYHA functional class and echocardiographic parameters improved over time. Statistical analysis demonstrated that root replacement technique and adult age were protective factors for SVD. We found no statistically significant difference in the clinical outcomes analyzed between women of childbearing age who had children after surgery and the rest of the women. The cryopreserved allograft is still a valid option in aortic valve replacement, providing acceptable durability and clinical outcomes with optimal hemodynamic performance. SVD is influenced by the implantation technique. Women of childbearing age might have additional benefits from this procedure.

## 1. Introduction

The consideration of cryopreserved aortic homograft (CAH) valve substitutes has been advocated as an acceptable alternative to conventional aortic valve replacement in selected patients [1,2]). Since their introduction by Sir Donald Ross in 1962, cryopreserved homografts have been widely used in the past in light of their advantages, inherent optimal hemodynamic performance, low thrombogenicity, avoidance of life-long anticoagulation, low rate of neurological events, and low risk of infection. In addition, they may also be used in patients contemplating pregnancy [3]. However, the initial enthusiasm has been tempered by issues regarding the durability of the cryopreserved substitutes with a reported incidence of structural degeneration (SVD) of more than 30% and a median time to reoperation for SVD ranging from 11 years in 0–25-year-old patients to 25 years in patients older than 50 [4]. Moreover, issues regarding the demanding surgical technique of implantation, limited availability, and increased complexity of reoperations have restricted its use, and there have been concerns about indications for its applications [5]. Although still under debate, there is consensus for its use in patients with acute endocarditis and periannular abscess and in women during pregnancy or of childbearing age [3,4,5,6,7]). We retrospectively reviewed our series of 210 patients who had undergone aortic allograft implantation with up to 20 years follow-up. The aim of our study was to identify factors influencing patients’ survival and durability of the aortic allograft, alongside identifying subgroups of patients with improved long-term quality of life, survival, and freedom from SVD.

## 2. Methods

### 2.1. Study Design and Oversight, Patient Population, Definitions, and Endpoints

Data were gathered from 210 patients between January 1993 to August 2010 and analyzed retrospectively. The databases were continuously monitored and audited by clinical information analysts within each unit and were validated periodically with internal checks. The study protocol has been registered at clinicaltrials.gov. (ClinicalTrials.gov ID: NCT05253469). The option of aortic valve replacement with cryopreserved homografts was primarily assessed using stringent inclusion criteria: young age, bacterial endocarditis recurrence, current or future pregnancy intentions, and contraindication to anticoagulation.

Primary endpoints included overall mortality, cardiac-related mortality, structural valve degeneration, and reoperation for valve-related diseases. Secondary endpoints included the cumulative incidence of adverse events (MACCEs, major adverse cardiac and cerebrovascular events), comparison of echocardiographic data, and specifically, outcomes in women of childbearing age.

Based on previous surgical experience and previously identified risk factors [8], patients were grouped according to their age (less than 25 years old, between 25 and 50 years old, and more than 50 years old), the technical procedure of allograft implantation (freehand vs. miniroot), and etiology of aortic valve disease (endocarditis, rheumatic, congenital, other) to elucidate potential subgroups in which the use of allograft might be more beneficial. This study was approved by the institutional review board (Approval Number assigned by the IRB: IRB MTP_2022_03_202201054). Patient consent was waived following the research guidance. This study complies with the Declaration of Helsinki.

### 2.2. Surgical Technique

**Figure 1:**
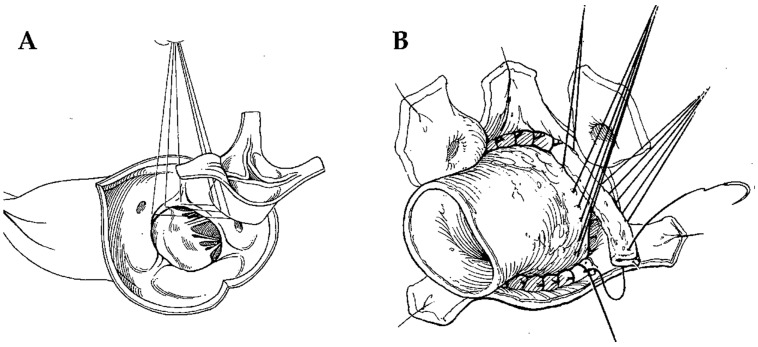
(**A**) subcoronary implantation; (**B**) miniroot implantation.

**Figure 2:**
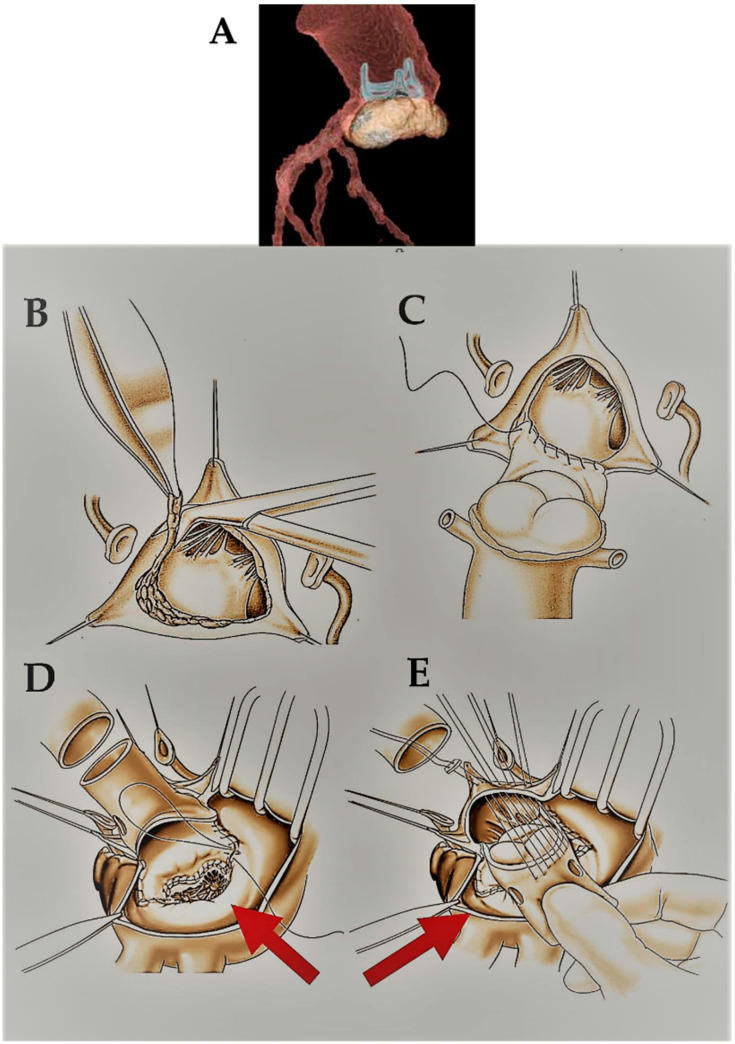
(**A**) Three-dimensional CT Scan reconstruction of PVE with extended periannular abscess. (**B**,**C**) A homograft is used for aortic root reconstruction and for the repair of mitro-aortic curtain using the miniroot procedure. The infected prosthesis is removed with aggressive debridement of all infected and necrotic tissue. The coronary ostia are prepared for the reconstruction of the aortic root. (**D**,**E**) Aortic and mitral homograft. Mitro-aortic endocarditis with the aortomitral curtain was largely involved. The abscess cavity is precisely bounded and debrided. A double homograft was used for the reconstruction (red arrows).

The technical details of aortic allograft insertion have been previously described [8]. The surgical strategy was based on the extent of the valvular lesions. Briefly, two techniques have been used: the freehand subcoronary implantation technique and the allograft root replacement with coronary reimplantation (miniroot) (Figure 1A,B). In the first, the homograft was scalloped with only the valve tissue and annulus grafted with a proximal interrupted suture line on the annulus and a distal running suture line on the ascending aorta. In the miniroot technique, the entire complex constituted by the aortic valve and sinuses of Valsalva was transplanted. The proximal anastomosis on the annulus was initially achieved with interrupted sutures, and then the allograft was positioned at the level of coronary ostia and secured with knots. Subsequently, the coronary arteries were reimplanted in situ and the distal anastomosis with the ascending aorta was completed with running sutures. The miniroot technique was a longer procedure due to the preparation time of the anastomosis on the two coronary buttons (Figure 1 and Figure 2).

### 2.3. Clinical Follow-Up

Periodical data collection was performed at 6 months and 1 year. In addition, cross-sectional analysis in a retrospective manner was carried out over the second half of the year 2018. Clinical checks of the patients were performed in the outpatient clinic or by phone interviews to obtain data for follow-up. Complete clinical examinations from the referring cardiologists or general practitioners were also accepted.

### 2.4. Echocardiography

Standard 2D and Doppler echocardiographic examinations with color-flow mapping were performed serially on all patients 1 week before the operation. Baseline echocardiography for clinical follow-up was performed immediately after the operation at the time of discharge. Follow-up echocardiography was obtained at the latest periodical exam or immediately before redo surgery. 

### 2.5. Structural Valve Degeneration

Structural valve degeneration was defined according to the guidelines as intrinsic changes to the allograft, such as calcification, tear, or other abnormality leading to dysfunction (stenosis or regurgitation exclusive of thrombosis or infection) [8,9,10,11,12]. Early technical failure and endocarditis were excluded. Diagnosis of structural degeneration relied on the aspect of the valve at reoperation and on echocardiographic surveillance. Leaflet thickening/calcification together with severe dysfunction (regurgitation grade 3–4, mean gradient > 19 mmHg, and/or valve area < 1 cm^2^) were considered echocardiographic findings of SVD.

### 2.6. Statistical Analysis

Categorical variables are expressed as frequencies and percentages. They were compared using the Chi-squared test or Fisher’s exact test, as appropriate. Continuous variables were checked for normality using the Kolmogorov–Smirnov test. Normally distributed variables are shown as mean and standard deviation and compared with parametric tests (Student’s *t*-test). Nonparametric variables are presented as median and interquartile range and compared using the Mann–Whitney test. Repeated measure variables were compared using the ANOVA test or Kruskal–Wallis test, with post hoc comparisons, as appropriate. Survival analyses were performed using the Kaplan–Meier analysis, and survival functions between groups were evaluated using the Log Rank test. Cox regression analysis was used to elucidate the role of preoperative and intraprocedural variables in the determinism of SVD. Variables were included in the multivariable model if their univariate *p* values < 0.2, using a sequential forward stepwise approach. Models were compared using the likelihood ratio test. The presented model best fits available data, consisting of 4 variables evaluated with 210 observations, and had an AUC of 0.777 and a Hosmer–Lemeshow *p* value of 0.985 (Chi-square with 8 df = 1.85), thus allowing us to assume its reliability. The proportional hazard assumption was checked both numerically and graphically. A two-tailed *p*-value < 0.05 was assumed statistically significant. Statistical analysis was executed with Stata/SE ver.13 for Windows (StataCorp LP, College Station, TX USA).

## 3. Results

### 3.1. Patients and Surgical Data

During the study period, 210 patients (125 males, 85 females) underwent aortic homograft valve replacement. The mean age was 40.1 ± 17.9 years (range 10–77 years) with 10 patients aged <18 years. The mean follow-up was 12.6 years, the median follow-up was 13.7 years, and the longest follow-up was 21.4 years. The rationale for choosing an aortic allograft was as follows: age less than 25 years (n = 42), endocarditis (n = 101), redo surgery (n = 48), women of childbearing age (n = 44), and contraindication to oral anticoagulation (n = 54). Miniroot allograft replacement with reimplantation of coronary arteries was performed in 155 cases, while the freehand technique was performed in 55 patients. Baseline characteristics and operative data are shown in Table 1.

### 3.2. Overall Mortality

**Figure 3:**
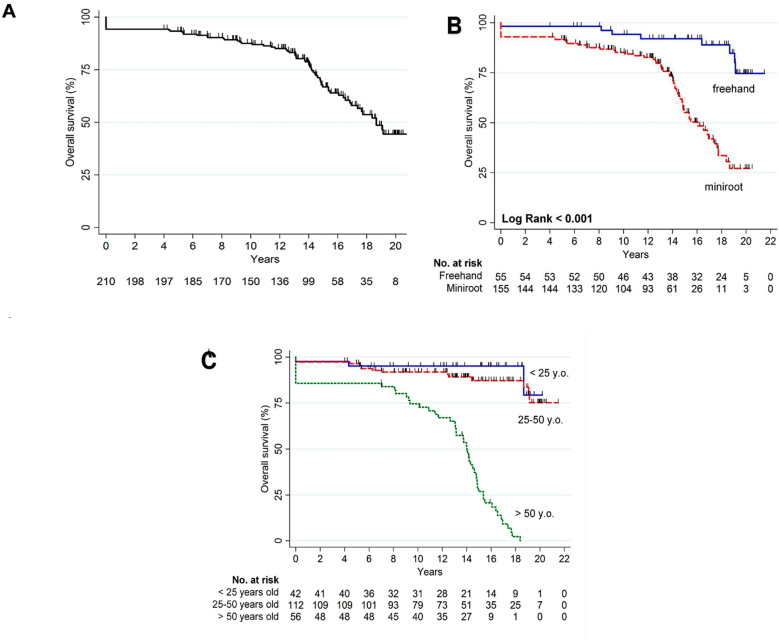
(**A**) Overall survival of the entire population; (**B**) overall survival between surgical techniques; (**C**) overall survival among age groups.

Figure 3A displays the Kaplan–Meier survival curve of the entire population. Overall mortality was 32.4% (68 events). Major non-cardiac causes of death occurred in 9.5% of the patients and were comprised of respiratory distress, chest infection, renal failure, hematological disorders, and cancer. Regarding the surgical technique, a statistically significant improved survival was found in the freehand group in comparison to the miniroot (8 events in 55 patients versus 60 events in 155, *p* < 0.001 by Log Rank test) (Figure 3B). This finding might be related to the higher number of endocarditis patients treated with the miniroot technique, who showed a poorer prognosis, as described below (35 in 55 patients in the freehand group and 66 in 155 in the miniroot group, *p* = 0.007). Further subgroup analysis showed a statistically significant difference in mortality in patients >50 years old (50 events in 56 patients) compared to the 25–50 and 0–25 years subgroups (*p* < 0.001 by Log Rank test). No significant difference was found among the other two groups (*p* = 0.432 by Log Rank test) (Figure 3C).

Early mortality was significantly increased in older patients and in redo procedures (*p* = 0.005 and <0.001, respectively) Table 2.

### 3.3. Cardiac Mortality Related to Structural Valve Degeneration

**Figure 4:**
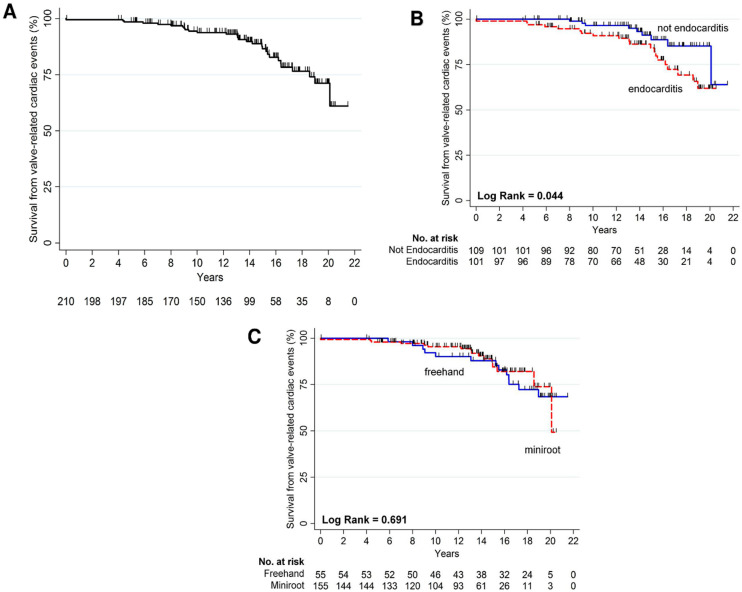
(**A**) Freedom from valve-related cardiac events; (**B**) survival from valve-related cardiac events compared using preoperative etiology; (**C**) freedom from valve-related cardiac events compared using surgical techniques.

SVD-related death occurred in 29 patients (13.8%), as shown in Figure 4A. Subgroup analysis demonstrated a statistically significantly higher incidence of endocarditis etiology in patients experiencing SVD-related death with respect to other etiological subgroups (*p* = 0.044 by Log Rank test, Figure 4B). No differences were found in SVD-related death compared by the surgical technique used (Figure 4C) and the age subgroups (Table 2).

### 3.4. Structural Valve Degeneration

SVD occurred in 57 patients (27.1%) with a statistically significantly higher incidence in the 0–25-year-old subgroup (Table 3). Additionally, the freehand subgroup was associated with a statistically significant increase in the occurrence of SVD (*p* < 0.001, Table 3). However, no differences among the etiology group could be detected (*p* = 0.642). Multivariable Cox regression showed that the miniroot technique and adult age were protective factors in SVD determination. Additionally, a higher allograft dimension was protective in SVD, but this result lacked statistical significance in multivariable analysis, probably because of the limited sample size (Table 4).

### 3.5. Reoperation

**Figure 5:**
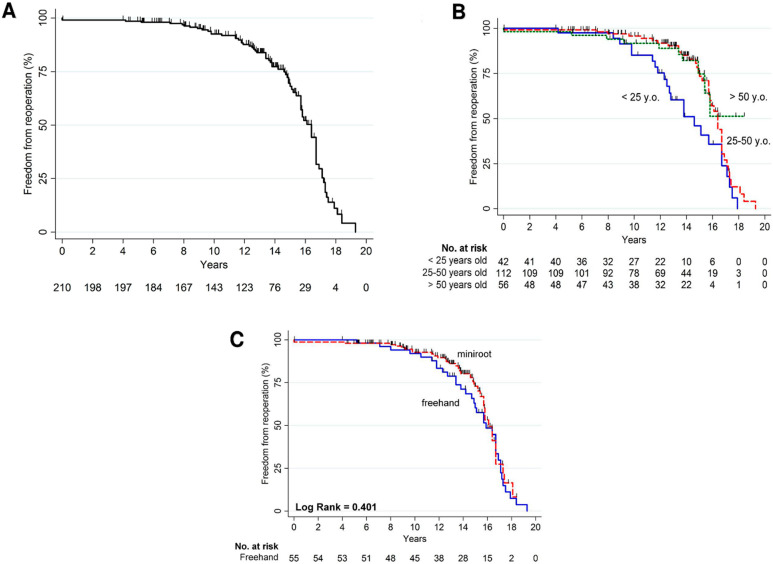
(**A**) Freedom from reoperation (overall); (**B**) freedom from reoperation according to age groups; (**C**) freedom from reoperation according to surgical techniques.

The Kaplan–Meier curve displaying freedom from reoperation is reported in Figure 5A. Seventy-one (33.8%) patients needed reoperation (Table 3) with 62% of the patients receiving a new aortic prosthesis and 18.3% an allograft, while 16.9% underwent a Bentall procedure and 2.8% an ascending aorta replacement. However, reoperation was found to occur more frequently in the younger subgroup of <25 years (*p* < 0.001, Table 3, Figure 5B). The surgical technique used and indication for surgery did not significantly influence the occurrence of reoperation (Figure 5C, Table 3).

### 3.6. Composite Cardiac End Point and Echocardiographic Data

**Figure 6:**
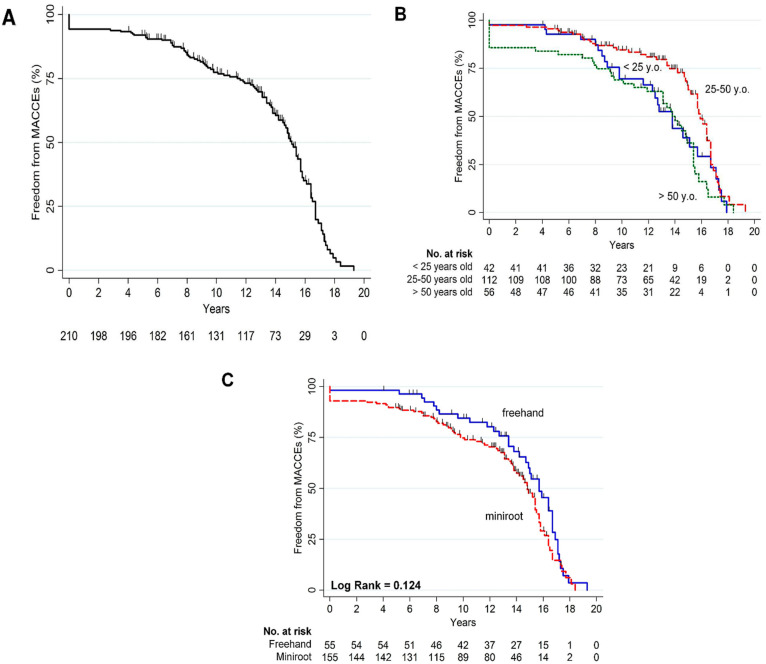
(**A**) Freedom from major adverse cardiac and cerebrovascular events (MACCEs) in the overall cohort; (**B**) MACCEs according to age groups; (**C**) MACCEs according to surgical techniques.

The cumulative Kaplan–Meier curve for freedom from MACCE occurrence is reported in Figure 6A. Major adverse cardiac and cerebrovascular events occurred in 115 patients (54.8%). Among these, older patients exhibited a significantly higher incidence of events (Table 3, Figure 6B). No other differences regarding the other subgroups analyzed were detected (Table 3, Figure 6C). Functional status as evaluated by NYHA class showed a statistically significant improvement both postoperatively and long-term in comparison to the baseline conditions. This improvement was stable over the course of the study, while the percentage of patients experiencing worsening NYHA class or rehospitalization for heart failure was 2.4% over the first year, 2.0% from the first to the fifth year, and 8.0% until the tenth year of follow-up, respectively (Table 3). Echocardiographic parameters are reported in Supplementary Table S1 and show that the surgical procedure promotes positive ventricular remodeling which remains stable over time.

### 3.7. Outcomes in Childbearing Age

In the overall population, there were 44 (21%) women of childbearing age. Among those, 37 (84.1%) patients became pregnant post-operatively and gave birth to normal children (Supplementary Table S2). We found no statistically significant difference in the clinical outcomes analyzed between women who had children and women who did not. Additionally, no differences were found between women who became pregnant and other women in our cohort with regard to clinical outcomes (Supplementary Table S2).

## 4. Discussion

This was a retrospective study of a large cohort of patients undergoing aortic valve replacement with a cryopreserved allograft with up to 20 years of follow-up. The main findings of this study were: (1) age > 50 years is associated with reduced long-term survival; (2) long-term SVD occurs in 27% of cases and is more frequent in young patients; (3) the miniroot replacement technique and adult age are protective factors for SVD; (4) endocarditis etiology determines an augmented risk of death related to allograft degeneration; (5) women of childbearing age might benefit from allograft implantation, as the long-term clinical outcomes are not different from the rest of the population.

The use of cryopreserved allograft for aortic valve and aortic root replacement has been shown to be a valid alternative in selected patients with aortic native or prosthetic valve endocarditis [1,2,6,8]. Unfortunately, the advantage inherent to the optimal hemodynamic performance and the avoidance of life-long anticoagulation is counterbalanced by the known tendency of these conduits to deteriorate, requiring reoperation normally associated with a high morbidity and early mortality of 8–17.9% [9]. However, recent reports claimed a 5-, 10-, and 15-year freedom from reoperation after aortic root implantation for acute native valve endocarditis of 83.7, 77.6, and 73.9%, respectively [6], and a large cohort study of 840 patients reported a 34% reintervention rate with an only 2% early mortality after reoperation [6]. In our cohort, reoperation occurred in 33.8% of the patients with an SVD incidence of 27%, mirroring the results of larger similar studies [8]. A comparison of these data with the outcome of the currently used biological glutaraldehyde-fixed xeno-tissue prosthetic valves, which are known to structurally deteriorate over an average of 10–20 years [10,11], basically supports the idea that cryopreserved allografts do not have a much shorter lifespan [2,4,8]. Conversely, homografts might provide patients with better hemodynamics, as transvalvular gradients are significantly lower, and the phenomenon of patient–prosthesis mismatch, known to afflict the conventional stented bioprostheses [8,12], rarely occurs after homograft implantation [13]. The intraoperative risk and the surgical challenges of a redo operation remain a daunting prospect, but the modern advancement in transcatheter technologies is a valid resource in this context, allowing for a less invasive resolution of the drawbacks related to allograft degeneration. However, considering the scarce knowledge of the long-term results of transcatheter valve implantation, especially in young patients, the use of sutureless valves has recently been suggested as an easier way to deal with redo allograft operations [14,15].

The present study demonstrates the benefit of homograft prostheses in the setting of infective endocarditis. The homograft option is dictated by a theoretical and practical principle. Fundamental surgical principles advocate the use of allogeneic tissue rather than the implantation of prosthetic material into an infected area, in the interest of minimizing the risk of recurrent infection. By choosing the homograft, the surgeon limits the prosthetic material solely to the sutures themselves. Based on these principles, aortic allografts have been advocated in the past as an alternative to extra-anatomical reconstruction for the repair of infected thoracic or descending abdominal aortic polyester grafts [16]. From an empirical technical standpoint for surgeons who have gained experience in handling cryopreserved homograft tissue, this valve substitute provides apparent flexibility in accommodating difficult root anatomy after aggressive debridement of infected tissue [6,7,8,9,10,11,12,13,14,15,16,17,18,19]. In fact, in the presence of active IE, the complete debridement of the septic tissue represents a fundamental stage that can lead to loss of heart structure, sometimes due to the demolition of large portions of infected tissue in most extensive lesions, which precedes the surgical momentum of reconstruction [6,7,8,20,21,22]. In patients in whom a conventional mechanical or stented xenograft valve prosthesis is chosen, a bovine pericardial patch or polyester-woven grafts can be used in conjunction with valve substitutes [5,7,18]. However, in using a homograft which is a flexible conduit, the anterior mitral valve leaflet can be employed to repair abscess cavities or other tissue defects. This procedure may be achieved either in an orthotopic position or by rotation of the homograft, depending on the characteristics of the lesion presented to the surgeon. In addition, in circumferential annular abscesses with partial or complete aorto-ventricular disruption, occurring particularly in prosthetic valve endocarditis, the entire inflow homograft can be sutured directly to the left ventricular outflow tract of recipients [7,8,20,21,22,23]. For surgeons who have not gained a good deal of experience with the use of homografts, the flexibility of the conduit may indeed pose technical challenges and therefore present a disadvantage [24].

In this study, we confirmed the importance of variables such as age and endocarditic etiology as factors for poor outcomes [2,3,5,7,12,13,14,15,19,25], but we found that endocarditis was able to statistically affect only the mortality due to SVD and did not produce a significantly different trend when the curve of the overall mortality was considered (Figure 4). This finding might be related to the underlying systemic conditions characterizing the other etiologies considered, such as rheumatic disease, which had a higher incidence of non-cardiac causes of death (kidney injury, lung disease, etc.), or could be associated with the local myocardial and endocardial environment induced by the endocarditis process, which created a hostile environment for the implant and the biological engrafting of the conduit. In this context, the implantation technique plays a fundamental role in strongly influencing the amount of tissue in contact with the host and therefore the susceptibility to immune reactions [6,7,8,20,21,22,23] and the geometry of the root complex after the replacement [4]. The miniroot technique is thought to preserve the aortic root geometry, minimizing aortic regurgitation [7,8,20,26] with greater durability than the subcoronary approach [1,2,4,8,27]. However, we suggest removing the excess tissue from the homograft to avoid geometrical obstruction and unnecessary tissue contact with the host [8,17,22,23,25]. In our study, we observed significantly improved survival with the freehand subcoronary approach, but this finding might be biased by the significantly higher percentage of patients with endocarditis, which are known to have worse outcomes in the miniroot group [8,17,22,23,25]. Conversely, we found that the freehand subcoronary technique was statistically associated with increased SVD compared to the root replacement technique for implanted allografts of similar sizes, confirming the results of other groups [27]. We might reliably speculate that the subcoronary technique might have increased tissue stress at the level of the valve leading to early SVD. Additionally, despite some advocating for reduced mechanical stress in the subcoronary technique because of the direct support of the aortic annulus [28], the difficulty in achieving a perfect orientation and cusp alignment during surgery might result in some degree of insufficiency, eventually leading to flow perturbation and the risk of SVD [2,4,8]. 

To further elucidate predisposing factors determining SVD, we performed a Cox multivariable analysis. Interestingly, the root replacement technique and adult age were protective factors in the development of SVD (Table 4). Of note, the size of the homograft, normally considered an important determinant in the hemodynamic outcomes after aortic valve replacement, failed to reach statistical significance in the multivariable model, but higher allograft dimensions were associated with less SVD during univariate analysis, reliably indicating that the limited sample size was underpowered to denote the potential significance of this variable in the model. Echocardiographic findings showed a stable improvement in transvalvular gradient, left ventricle diameters, and ejection fraction. These data contributed to a stable improvement in NYHA class in most of the patients in the long term. 

As a final remark, 84% of the women of childbearing age gave birth to one or more babies, and we found no difference in the clinical outcomes in these women compared to the rest of the women who underwent surgery. This result is surprising as it is discordant with previous findings on mitral homografts [8] in which women with homografts developed SVD some years after delivery and on bioprostheses [3,29,30,31]. In this regard, the imbalance in estro-progestinic equilibrium or other hemodynamic conditions occurring during pregnancy might play a role in the degeneration of the graft [3,29,30]. However, in the current study, we did not observe any differences in the outcomes, indicating that the aortic allograft, in avoiding anticoagulation, might constitute an additional advantage for this subcategory of patients by permitting a ‘normal life’ after the operation [8].

Finally, the Ross procedure is rarely adopted in IE. The increased surgical complexity alongside the potential for long-term failure of 2 valves (aortic and pulmonary) has been discouraging with a 3-fold increase in operative mortality compared to conventional aortic valve replacement. However, a volume–outcome relationship has been reported with lower mortality in high-volume centers (0.3–1.1%) [32,33]. This rekindled the role of the pulmonary autograft in the management of aortic valve endocarditis, when avoiding prosthetic material is necessary, when there is increased risk of relapsing infection, or in women of childbearing age. The Ross operation has shown optimal long-term results with low rates of valve-related complications for recurrent endocarditis. Patients with a life expectancy > 15 years, an active lifestyle, and no severe comorbidities should be referred to centers with high surgical experience [32,33,34,35].

## 5. Limitations

The authors acknowledge the typical limits related to the retrospective nature of the study, and therefore, mechanistic relationships between exposure variables and outcomes are difficult to evaluate. Additionally, considering the wide timeframe of analysis, adjustments for changes in surgical management or technique, risk factors, or surgical experience should have been performed. However, considering the relatively short time range in which the operations have been performed, we can reliably speculate that these confounding variables would not have played a role in the results of the study. A time-based analysis was performed by Fukushima et al. in their series of 840 patients, with a timeframe of analysis significantly wider than in our cohort (from 1975 till 2008) [1], in which drastic changes in healthcare occurred. Another limit consists of the presence of censored cases in the survival analysis that might have underpowered the statistical significance of the analysis. However, this point might also relate to the shorter follow-up of patients operated on in recent years of the study. Thirdly, the fact that some of the long-term data on the clinical and echocardiographic follow-up have been obtained by other medical institutes or cardiologists without the original institute in which the patients were operated on and followed-up might have introduced a potential bias. For this reason, we have not conducted a complete analysis of the long-term echocardiographic findings due to the potential variation in the reliability of the results. Similarly, the echocardiographic data of donors would have been interesting but were not available. As for the data on the clinical outcomes, such as death or adverse events, those were confirmed in every case with death certificates and medical charts and, therefore, can be considered reliable.

## 6. Conclusions

Cryopreserved allografts are still a valid option in aortic valve replacement, providing acceptable durability and clinical outcomes with optimal hemodynamic performance. They may also be additionally beneficial for women of childbearing age.

However, evidence about the use of human biological substitutes is conflicting [7]. The wide differences in the clinical characteristics of the patients undergoing aortic valve replacement with allograft (etiology, age, previous surgery, and operative techniques) would require a collaborative metanalysis in an attempt to reduce the impact of these confounding variables. Considering the difficulty in performing an actual randomized clinical trial, such an analysis would be crucial to dissipate doubts regarding the more appropriate use of allografts and to determine the categories of patients who would majorly benefit from this procedure.

## 7. Perspectives

### 7.1. Competency in Medical Knowledge

A cryopreserved allograft is still a valid option in aortic valve replacement by providing acceptable durability and clinical outcomes with optimal hemodynamic performance.

### 7.2. Competency in Patient Care

Specific subgroups of patients, such as young adults, endocarditis patients, and women of childbearing age, should be offered cryopreserved allograft aortic valve replacements and be adequately counselled on this surgical option, clarifying the potential risk for valve degeneration and reoperation but also the benefit arising from optimal hemodynamic performance, avoidance of life-long anticoagulation, and the possibility of having children (women of childbearing age).

### 7.3. Translational Outlook

Despite its limitations, the result of this study might provide help to cardiologists in discussions about the risks, benefits, and expectations after cryopreserved homograft aortic valve replacement. Additionally, from these data, we can hypothesize that improvement in the methods of homograft preservation might prolong its durability and provide an additional stimulus for its usage.

## Figures and Tables

**Table 1:** Baseline characteristics and operative data.

Baseline Characteristics	Patients
Patients	210 (100%)
Male sex	125 (59.5%)
Mean age (range)	40.1 ± 17.9 (10–77)
Age groups	
<25 years	42 (20.0%)
25–50 years	112 (53.3%)
>50 years	56 (26.7%)
Smoking history	21 (10.0%)
Hypertension	24 (11.4%)
Dyslipidemia	6 (2.8%)
Diabetes	4 (1.9%)
Chronic obstructive pulmonary disease	12 (5.7%)
Chronic kidney disease	5 (2.4%)
Preoperative NYHA class	
1	0 (0.0%)
2	64 (30.5%)
3	83 (39.5%)
4	63 (30.0%)
Etiology	
Endocarditis	101 (48.1%)
<25 years	27
25–50 years	67
>50 years	7
Rheumatic	57 (27.1%)
<25 years	13
25–50 years	24
>50 years	20
Congenital	35 (16.7%)
<25 years	2
25–50 years	16
>50 years	17
Other	17 (8.1%)
<25 years	0
25–50 years	5
>50 years	12
Surgical indication	
Isolated aortic stenosis	46 (21.9%)
Isolated aortic regurgitation	85 (40.5%)
Mixed aortic stenosis and regurgitation	79 (37.6%)
Previous cardiac surgery	48 (22.8%)
Aortic valve replacement, mechanical	23 (10.9%)
Aortic valve replacement, biological	13 (6.2%)
Homograft	10 (4.8%)
Other	2 (0.9%)
Repeated cardiac surgery	48 (22.8%)
Redux	42 (20.0%)
Tridux	5 (2.4%)
Quadridux	1 (0.5%)
**Operative Data**	**Patients**
Concomitant procedure	49 (23.3%)
Myocardial revascularization	19 (9.0%)
Mitral valve homograft	10 (4.8%)
Tricuspid valve repair	9 (4.3%)
Mitral homograft valve repair	8 (3.8%)
Tricuspid valve replacement	3 (1.4%)
Technical procedure	
Freehand	55 (26.2%)
By etiology	
Endocarditis	35
Rheumatic	10
Congenital	8
Other	2
By age	
<25 years	16
25–50 years	34
>50 years	5
Miniroot	155 (73.8%)
By etiology	
Endocarditis	66
Rheumatic	47
Congenital	27
Other	15
By age	
<25 years	26
25–50 years	78
>50 years	51
Allograft mean size	
Freehand, mean-SD/median-IQR	22.6 ± 1.5, 22 (20–24)
Miniroot, mean-SD/median-IQR	22.5 ± 1.8, 22 (20–24)
Severe aortic annular calcification	32 (15.2%)
Donor age (range)	44.1 ± 7.1 (5–61)
Donor age > 50 years	68 (32.3%)
Donor annulus, mean-SD/median-IQR	25.2 ± 3.6, 25 (22–28)
Gender mismatch	92 (43.8%)
Annular size mismatch (>5 mm)	11 (5.2%)
Blood group mismatch	109 (51.9%)
Rh antigen mismatch	34 (16.2%)

**Table 2:** Clinical outcomes.

Outcome	Patients	*p* Value
Early mortality	12 (5.7%)	
By age		0.005 ^y^
<25 years	1 (2.4%)	
25–50 years	3 (2.7%)	
>50 years	8 (14.3%)	
By etiology		0.165
Endocarditis	9 (8.9%)	
Rheumatic	3 (5.3%) *	
Congenital	0 (0%)	
Other	0 (0%)	
By technical procedure		0.147
Freehand	1 (1.8%)	
Miniroot	11 (7.1%)	
By cardiac procedure		<0.001 ^z^
First	7 (4.3%)	
Second	2 (4.8%)	
Third	2 (40.0%)	
Fourth	1 (100.0%)	
Overall mortality	68 (32.4%)	
By age		
<25 years	3 (7.1%)	<0.001 ^x^
25–50 years	15 (13.4%)	
>50 years	50 (89.3%)	
By etiology		
Endocarditis	22 (21.8%)	<0.001 ^jj^
Rheumatic	22 (38.6%)	
Congenital	16 (45.7%)	
Other	8 (47.0%)	
By technical procedure		
Freehand	8 (14.5%)	<0.001
Miniroot	60 (38.7%)	
Valve related cardiac mortality	29 (13.8%)	
By age		
<25 years	3 (7.1%)	0.276
25–50 years	15 (13.4%)	
>50 years	11 (19.6%)	
By etiology		
Endocarditis	20 (19.8%)	0.044
Rheumatic	6 (10.5%)	
Congenital	2 (5.7%)	
Other	1 (5.9%)	
By technical procedure		
Freehand	5 (9.1%)	0.691
Miniroot	24 (15.5%)	
Not valve related cardiac mortality	19 (9.0%)	
Not cardiac mortality	20 (9.5%)	

^y^ Early mortality is greater with increasing age. ^z^ Early mortality is greater in redo, 3rd time, and 4th time procedures. ^x^ Patients aged <25 years have a reduced overall mortality compared to other groups. ^jj^ Patients with endocarditis have a reduced overall mortality compared to other groups. Patients with endocarditis have an increased valve-related cardiac mortality compared to other groups. * Two of these patients have signs of inactive endocarditis.

**Table 3:** Structural valve degeneration, reoperation, freedom from MACCEs, and NYHA class.

Outcome	Patients	*p* Value
Structural valve degeneration By age <25 years 25–50 years >50 years By etiology Endocarditis Rheumatic Congenital Other By technical procedure Freehand Miniroot	57 (27.1%) 22 (52.4%) 30 (26.8%) 5 (8.9%) 31 (30.7%) 15 (26.3%) 7 (20.0%) 4 (23.5%) 27 (49.1%) 30 (19.3%)	<0.001 * 0.642 <0.001
Reoperation By age <25 years 25–50 years >50 years By etiology Endocarditis Rheumatic Congenital Other By technical procedure Freehand Miniroot Reoperation type Aortic valve replacement (mechanical) Aortic allograft Bentall procedure Aortic valve replacement (biologic) Ascending aorta	71 (33.8%) 23 (54.8%) 38 (33.9%) 10 (17.8%) 38 (37.6%) 19 (33.3%) 9 (25.7%) 5 (29.4%) 34 (61.8%) 37 (23.9%) 36 (50.7%) 13 (18.3%) 12 (16.9%) 8 (11.3%) 2 (2.8%)	0.001 ^y^ 0.787 0.401
MACCEs By age <25 years 25–50 years >50 years By etiology Endocarditis Rheumatic Congenital Other By technical procedure Freehand Miniroot	115 (54.8%) 26 (61.9%) 49 (43.7%) 40 (71.4%) 51 (50.5%) 31 (54.4%) 21 (60.0%) 12 (70.6%) 36 (65.4%) 79 (51.0%)	<0.001 ^z^ 0.914 0.124
NYHA class Preoperative (mean ± SD) 1 2 3 4 After 6 months from surgery (mean ± SD) 1 2 3 4 After 1 year from surgery (mean ± SD) 1 2 3 4 After 5 years from surgery (mean ± SD) 1 2 3 4 After 10 years from surgery (mean ± SD) 1 2 3 4	3.0 ± 0.8 0 64 83 63 1.4 ± 0.5 127 66 5 0 1.3 ± 0.5 144 51 2 1 1.3 ± 0.5 147 47 3 1 1.4 ± 0.6 97 50 1 2	<0.001 ^x^ 0.002 ^x^ 0.562 ^x^ <0.001 ^x^

MACCEs: major adverse cardiac and cerebrovascular events. SD: standard deviation. * Younger patients have an increased structural valve degeneration compared to other groups. ^y^ Patients aged <25 years have an increased reoperation rate compared to other groups. ^z^ Patient age > 50 years had a minor freedom from MACCEs compared to patients aged 25–50 years. ^x^ Compared to previous timepoint, using a nonparametric test for paired data.

**Table 4:** Univariate and multivariable Cox regression model on SVD.

Univariate Analysis			
Variable	Significance	Hazard Ratio	95%CI
Miniroot	<0.001	0.25	0.13–0.48
Age	<0.001	0.94	0.92–0.97
Endocarditis	0.166	1.41	0.77–1.60
Allograft dimension	0.027	0.82	0.68–0.98
Etiology Rheumatic Congenital Other	0.561 0.228 0.551	0.81 0.56 0.69	0.39–1.67 0.22–1.43 0.21–2.30
Male sex	0.094	1.73	0.91–3.30
Hypertension	0.228	0.50	0.16–1.54
Smoking history	0.074	0.26	0.06–1.14
Diabetes	0.923	0.89	0.09–8.76
**Multivariable Analysis**			
**Variable**	**Significance**	**Hazard Ratio**	**95%CI**
Age Miniroot Endocarditis Allograft dimension Constant	<0.001 0.001 0.222 0.249 0.043	0.95 0.30 0.64 0.89 89.89	0.92–0.97 0.14–0.62 0.31–1.31 0.43–1.08 1.16–6945.78

95%CI: 95% confidence interval for the hazard ratio.

## Data Availability

Drs. Nappi, Nenna, and Acar had full access to all the data in the study and take responsibility for the integrity of the data and the accuracy of the data analysis. The data underlying this article will be shared on reasonable request to the corresponding author.

## Supplementary Materials

The following supporting information can be downloaded at: https://www.mdpi.com/article/10.3390/jcdd10060248/s1, Table S1: Echocardiographic parameters; Table S2: Outcomes of women of childbearing age.

## Abbreviations

AUC	area under the ROC curve
CAH	cryopreserved aortic homograft
MACCEs	major adverse cardiac and cerebrovascular events
NYHA	New York Heart Association
SVD	structural valve degeneration
