# Supplementary material for: The Use of the Cryopreserved Aortic Homograft for Aortic Valve Replacement: Is It Still an Option?
Source: J Cardiovasc Dev Dis. 2023 Jun 8;10(6):248. doi: 10.3390/jcdd10060248 (PMC10299650; doi:10.3390/jcdd10060248)
Supplement: Supplementary file 1 [file jcdd-10-00248-s001.zip › jcdd-2350979-supplementary.pdf]

Supplementary Table S1. Echocardiographic parameters.

		LVEDD	LVESD	LVEF	AV regurgitation grade	AV mean gradient	Annular diameter	LVOT diameter	Aortic diameter
Discharge		46.9±4.7	36.2±6.1	57.3±5.5	0.4±0.5	9.0±0.8	22.4±1.9	21.9±1.9	34.1±4.1
Last follow up		46.3±4.7	35.8±6.0	58.0±5.8	0.8±0.7	9.9±2.0	22.5±1.7	21.9±1.9	33.9±4.9
P value		<0.001*	<0.001*	<0.001*	<0.001*	<0.001	0.008*	0.319	0.440
Age	< 25 years (a)	42.1±3.0	30.3±4.9	61.9±2.8	1.1±0.9	11.1±3.1	21.1±2.2	30.0±2.8	28.6±3.8
	25-50 years (b)	45.3±2.9	35.1±5.2	59.6±3.6	0.6±0.5	9.3±0.8	22.9±1.3	22.3±1.2	34.6±4.4
	> 50 years (c)	51.6±4.0	41.4±3.4	51.6±6.0	0.8±0.8	10.1±2.2	22.8±1.1	22.3±1.0	36.5±1.9
	P value (ANOVA)	<0.001[y]	<0.001[y]	<0.001[y]	0.015[y]	<0.001[y]	<0.001[y]	<0.001[y]	<0.001[y]
	P value (a-b)	<0.001	<0.001	<0.001	0.016	<0.001	<0.001	<0.001	<0.001
	P value (a-c)	<0.001	<0.001	<0.001	0.782	0.085	<0.001	<0.001	<0.001
	P value (b-c)	<0.001	<0.001	<0.001	0.282	0.103	1.000	1.000	0.028
Technique	Freehand	43.7±2.6	33.2±4.7	61.2±2.7	0.8±0.6	9.8±1.5	22.7±1.5	22.1±1.6	33.0±3.1
	Miniroot	47.4±5.0	36.9±6.3	56.5±6.2	0.8±0.8	9.9±2.2	22.4±1.7	21.7±2.0	34.3±5.2
	P value	<0.001[y]	<0.001[y]	<0.001[y]	0.711[y]	0.664[y]	0.372[y]	0.288[y]	0.118[y]
Allograft size	> 21 mm	44.3±5.3	32.2±6.8	59.4±6.9	1.0±0.8	11.1±2.7	20.6±1.2	19.8±1.8	31.5±4.6
	≤ 21 mm	47.3±4.1	37.6±4.7	57.2±5.0	0.7±0.7	9.2±1.1	23.4±0.8	22.9±0.6	35.1±4.2
	P value	<0.001[y]	<0.001[y]	0.025[y]	0.040[y]	<0.001[y]	<0.001[y]	<0.001[y]	<0.001[y]
Endocarditis	yes	47.7±5.1	37.3±6.1	56.1±6.6	0.8±0.7	10.0±2.1	22.5±1.6	22.0±1.6	34.2±3.6
	no	44.7±3.7	34.0±5.6	60.1±3.6	0.8±0.7	9.7±1.9	22.4±1.8	21.7±2.1	33.4±5.6
	P value	<0.001[y]	<0.001[y]	<0.001[y]	0.728[y]	0.290[y]	0.702[y]	0.447[y]	0.311[y]

Supplementary Table 1. Echocardiographic parameters. Abbreviations; AV, aortic valve; EF, ejection fraction (%) LVEDD; left ventricular end diastolic diameter (mm); LVESD, left ventricular end systolic diameter (mm). LVOT; left ventricle outflow tract; * paired data analysis; [y] unpaired data analysis at last follow-up

Supplementary Table S2. Outcomes of women in childbearing age.

Outcomes	Women in childbearing age who had children	Women childbearing age who did not have children	P value
Number of patients	37	7	
Overall death	17(45.9%)	5(71.4%)	0.216
Valve related cardiac death	4(10.8%)	0(0.0%)	0.362
Structural valve degeneration	9(24.3%)	2(28.6%)	0.812
Reoperation	10(27.0%)	2(28.6%)	0.933
MACCEs	20(54.0%)	5(71.4%)	0.395

Outcomes	Women in childbearing age who delivered after surgery	All ages women	P value
Number of patients	37	48	
Valve related cardiac death	4(10.8%)	7(14.6%)	0.607
Structural valve degeneration	9(24.3%)	5(10.4%)	0.087
Reoperation	10(27.0%)	9(18.7%)	0.364
MACCEs	20(54.0%)	19(39.6%)	0.184
